# Protocol for high-density peptide and protein presentation on biomaterial surfaces using virus-like particles

**DOI:** 10.1016/j.xpro.2026.104739

**Published:** 2026-07-23

**Authors:** Rayane Hedna, Hasna Maayouf, Thomas Dos Santos, Laurent Pieuchot

**Affiliations:** 1 Mulhouse Institute of Materials Science (IS2M), CNRS UMR 7361, University of Haute-Alsace, 68100 Mulhouse, France

**Keywords:** Cell culture, Protein expression and purification, Biotechnology and bioengineering

## Abstract

Here, we present a protocol for achieving high-density peptide and protein presentation on biomaterial surfaces using self-assembling virus-like particles. We describe steps for the design, recombinant production, purification, and quality control of peptide- or protein-displaying particles. We also detail procedures for their use in functionalizing polydimethylsiloxane (PDMS) and other solid substrates. The approach enables multivalent and spatially controlled ligand presentation and is applicable to reproducible cell-material interaction studies and the biofunctionalization of implant-relevant surfaces.

For complete details on the use and execution of this protocol, please refer to Maayouf et al.^1^

## Highlights

• Generate peptide- and protein-displaying virus-like particles
• Functionalize PDMS surfaces using AP205 and Mi3 VLP platforms
• Tune ligand density by SpyTag-SpyCatcher surface conjugation
• Enable cost-effective, high-copy, and multiligand surface functionalization

## Abstract (editor highlights)

Publisher’s note: Undertaking any experimental protocol requires adherence to local institutional guidelines for laboratory safety and ethics.

## Graphical abstract

**Figure:**
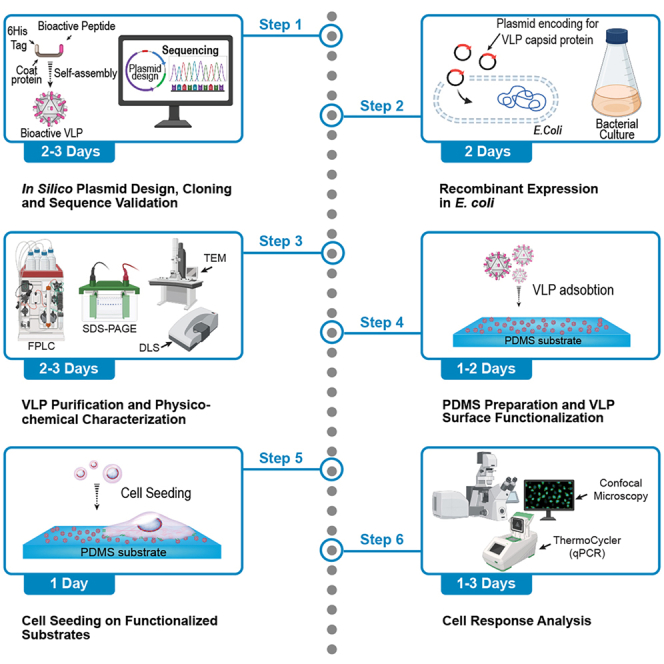

## Before you begin

This protocol outlines a streamlined workflow for generating biomaterial surfaces with high-density presentation of bioactive peptides or proteins using self-assembling virus-like particles (VLPs). It has been optimized for the functionalization of polydimethylsiloxane (PDMS) substrates using AP205 and Mi3 VLP platforms, enabling controlled ligand display for studies of cell-material interactions and broader tissue engineering applications. The workflow is adaptable to other solid substrates and VLP systems with appropriate adjustments and can be readily implemented in laboratories equipped for molecular cloning, recombinant protein expression in *E. coli*, and mammalian cell culture.

**Figure 1:**
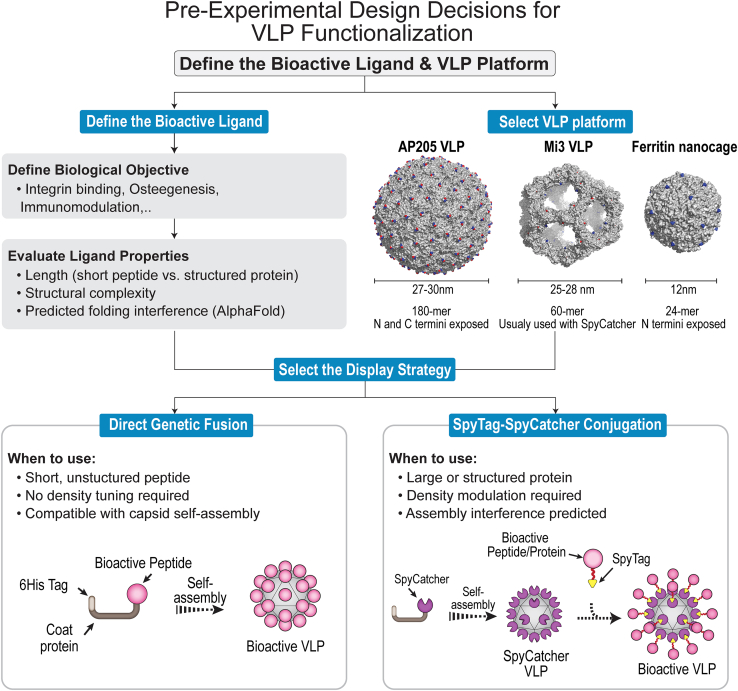
Pre-experimental design considerations for selecting virus-like particle (VLP) platforms and ligand display strategies This schematic outlines the key parameters to evaluate prior to initiating the protocol for engineering bioactive ligand–displaying VLPs. The design process begins with defining the biological objective and selecting an appropriate bioactive peptide or protein. Ligand properties such as length, structural complexity, and potential interference with capsid assembly should be assessed, including predicted structural compatibility using tools such as AlphaFold. In parallel, the choice of VLP platform determines the available valency and surface presentation geometry. Representative platforms include AP205 VLPs (∼27–30 nm diameter, 180 subunits), which provide high valency and multiple accessible termini for ligand display; Mi3 VLPs (∼25–28 nm diameter, 60 subunits), which support stable modular functionalization through SpyCatcher-based conjugation; and ferritin nanocages (∼12 nm diameter, 24 subunits), which form compact assemblies with surface-accessible N-terminal regions that can be used for ligand presentation. Based on ligand characteristics and the desired level of control over display density, either direct genetic fusion or SpyTag–SpyCatcher–mediated conjugation can be selected as the appropriate display strategy.

Before starting the protocol, users must define the bioactive peptide(s) or protein(s) to be displayed, select the corresponding VLP display strategy and the VLP system to be employed. Two display strategies are implemented in this protocol: direct genetic fusion of peptides to the VLP capsid protein, and post-assembly functionalization using SpyTag–SpyCatcher chemistry. The selected strategy determines the design of expression constructs and the subsequent steps of the protocol (see Figure 1).

1. Selection of the Bioactive Peptide or Protein
  a. **Biological relevance:** Choose a peptide or protein with well-characterized bioactivity in the desired context.
    ***Note:*** Examples include RGD peptide for integrin binding, BMP-2 mimetic sequences for osteogenesis, anti-inflammatory peptides for wound healing or anti-microbial peptides.
  b. **Peptide length:** Select short peptides sequence for genetic fusion to VLP subunits.
    ***Note:*** For genetic fusion to VLP subunits, short peptides (5–30 amino acids) are generally preferred to minimize interference with VLP assembly. Longer domains (>50 amino acids) or structured proteins require careful optimization and testing, as they may impair capsid formation or solubility.
  c. **Peptide and sequence features:** Evaluate sequence parameters such as hydrophobicity, net charge, secondary structure tendency, and the presence of repetitive or aggregation-prone motifs.
    **CRITICAL:** These features may affect protein expression, solubility, VLP assembly, and surface presentation efficiency.
  d. **Positioning of epitopes:** Determine whether the N- or C-terminus of the VLP subunit is more permissive to fusion.
    ***Note:*** For certain VLPs, such as MS2, peptide display can also be achieved by insertion into surface-exposed loop regions, depending on the size and structural constraints of the peptide. Literature on the VLP platforms (e.g., AP205, MS2, Qβ, HBc, or self-assembling proteins like ferritin or encapsulins) should be consulted to guide this decision.^2^^,^^3^^,^^4^^,^^5^

2. Selection of the VLP Platform.
  a. Select the VLP platform according to the desired ligand display strategy, particle geometry, structural stability, and downstream application.

***Note:*** Multiple VLP platforms may be used depending on the intended application. AP205 VLPs are highly stable, permissive to both N- and C-terminal fusions, and compatible with genetic display or SpyTag/SpyCatcher-mediated conjugation approaches. They are also widely used as a platform for vaccine development and vaccine research.^6^^,^^7^^,^^8^^,^^9^ Mi3 is an engineered dodecahedral VLP particularly suited for multivalent post-assembly functionalization through SpyCatcher display. Other systems, including MS2, Qβ, HBcAg, encapsulins, or ferritin, may also be used depending on the desired particle geometry, size, and expression system, although protocol optimization may be required.

3. Selection of the Display Strategy
  Two display strategies are supported in this protocol:
  a. Direct genetic fusion: Use direct genetic fusion for short and relatively unstructured peptides. **Note:** This strategy is suitable for short, unstructured peptides. The peptide is fused to the coat protein gene and expressed in E. coli. In AP205, the N-terminus could be used for histidine tag fusion, while the C-terminus can be used for peptide display. Co-display of two different peptides can be achieved using dual-expression plasmids or co-transformation of compatible vectors encoding different fusions.
  b. SpyTag–SpyCatcher-mediated conjugation: Use SpyTag–SpyCatcher-mediated conjugation for modularpost-assembly functionalization of peptides, full-length proteins, fluorescent proteins, or chemically modified ligands. In this strategy, VLPs are engineered to present SpyCatcher domains on their surface, and the ligand of interest is produced separately with a SpyTag.

***Note:*** SpyTag–SpyCatcher is a peptide–protein pair that forms a spontaneous, irreversible covalent bond under physiological conditions, enabling robust and modular post-assembly conjugation of ligands to VLPs. This allows tuning of ligand identity and density without altering the VLP scaffold. For background, see Zakeri et al.,^10^ Keeble et al.^11^

**CRITICAL:** The choice between genetic fusion and conjugation determines key properties such as ligand stoichiometry, display density, and flexibility. It must be made prior to construct design and cloning.

Once the bioactive peptide, the display strategy and VLP platform have been selected, users should compile a list of the required plasmids. When possible, existing plasmids can be sourced from repositories such as Addgene or requested from authors. If unavailable, constructs may be ordered as gene synthesis fragments for subcloning.

The remainder of this protocol is based on two case studies: (1) direct genetic fusion of one or two peptides (RGD and YIGSR) to the AP205 coat protein, and (2) post-assembly functionalization of SpyCatcher-presenting Mi3 VLPs with SpyTag-fused ligands, including RGD peptides, GFP, and mKate fluorescent proteins. The complete list of plasmids and their sources is provided in the materials and equipment section.

***Note:*** We are happy to share our plasmids with interested researchers upon request.

### Innovation

Conventional approaches to biomaterial surface functionalization typically rely on passive adsorption or chemical immobilization of peptides and proteins. These methods offer limited control over ligand density, orientation, and the spatial presentation of multiple signals. This protocol introduces a modular strategy based on virus-like particles (VLPs), which serve as self-assembling, nanoscale scaffolds for controlled ligand display at material interfaces.

The innovation lies in harnessing the intrinsic multivalency and defined geometry of VLPs to achieve reproducible, high-density, and spatially organized presentation of bioactive peptides or proteins that effectively engage cell surface receptors. By integrating two complementary display strategies, direct genetic fusion and SpyTag–SpyCatcher-mediated conjugation, the platform supports both fixed-stoichiometry display of short peptides and modular post-assembly functionalization with larger peptides or full-length proteins.

While the underlying techniques, molecular cloning, bacterial expression, and surface adsorption, are well established, their integration into a unified VLP-based platform for surface biofunctionalization represents a novel conceptual advance. This approach enables users to generate their own bioactive surfaces directly from recombinant constructs, offering a customizable and cost-effective alternative to commercial extracellular matrix proteins such as fibronectin.

It enhances experimental reproducibility, allows precise tuning of ligand identity and density, and supports a wide range of applications in cell–material interaction studies and tissue engineering.

### Institutional permissions

All experiments involving recombinant DNA and genetically modified microorganisms were conducted in accordance with institutional biosafety regulations and approved laboratory practices. Mammalian cell culture experiments were performed following standard institutional biosafety guidelines. No live vertebrate animals, higher invertebrates, or human subjects were involved in this study; therefore, institutional ethical approval was not required.

## Key resources table

**Table:** 

REAGENT or RESOURCE	SOURCE	IDENTIFIER
**Antibodies**		

Anti-6×His tag mouse monoclonal antibody (1:1000)	Abcam™	Ab18184
Goat Anti-Mouse IgG H&L (Alexa Fluor® 647) (1:500)	Abcam™	Ab150115
Alexa Fluor™ 488 Phalloidin (1:40)	Invitrogen™	A12379
Alexa Fluor™ 568 Phalloidin (1:50)	Invitrogen™	A12380
Hoechst 33342 (1:50)	ThermoFisher™	H3570
Rabbit monoclonal anti-vinculin antibody (1:300)	Sigma™	V9131
Goat anti-rabbit Alexa Fluor™ 488 (1:500)	Abcam™	Ab150077
Mouse anti-integrin β1 antibody (1:300)	Sigma™	I9018
Rabbit anti-αvβ3 antibody (1:200)	ThermoFisher™	BS-1310R
Goat anti-rabbit Chromeo™ 546 (1:500)	Abcam™	Ab60316

**Bacterial and virus strains**		

*E. coli* TOP10	Invitrogen™	C404003
*E. coli* BL21 Star™	Invitrogen™	C600003

**Chemicals, peptides, and recombinant proteins**		

IPTG	Sigma™	I6758
Lysozyme	Sigma™	L6876
DNase	Sigma™	D4263
RNase	Sigma™	R4642
Imidazole	Sigma™	I5513
LB broth base	Invitrogen™	12780029
Ampicillin	Sigma™	A9518
Kanamycin	Sigma™	K1637
RGD peptide	Selleck Chemicals	S8008
Laminin	Sigma™	CC095
Dulbecco’s modified Eagle’s medium (DMEM)	Gibco	11965092
Fetal Bovine Serum	Gibco	17994721

**Critical commercial assays**		

In-Fusion HD Cloning Kit	Takara Bio	638910
GeneJet Plasmid DNA Miniprep Kit	ThermoFisher™	K0503
Qiagen Gel Extraction Kit	Qiagen™	28704
CloneAmp™ HiFi PCR Premix	Takara Bio	639298
Micro BCA Protein Assay Kit	Thermo Scientific™	23235
Alamar Blue Cell Viability Reagent	Sigma™	DAL1025
Kit Taq'Ozyme OneMix w/Dye	Ozyme	OZYA011

**Experimental models: Cell lines**		

Mouse myoblast C2C12 cells	ATCC®	CRL-1772™

**Oligonucleotides**		

PCR primers for cloning	Maayouf et al^1^	Table 3, Table 4(This paper)

**Recombinant DNA**		

pET15b-AP205 constructs	Maayouf et al^1^	N/A
pETDuet-AP205 (coexpression constructs)	Maayouf et al^1^	N/A
pET28a-SpyTag003-mKate2 Plasmid	Addgene	#133452
pET28a-SpyTag003-sfGFP Plasmid	Addgene	#133454
pQE80L-SpyTag-ELP-SpyTag Plasmid	Addgene	#112634
pET15b-SpyTag-ELP-RGD	Maayouf et al^1^	N/A
pET28a-SpyCatcher003-mi3 plasmid	Addgene	#159995

**Software and algorithms**		

PyMOL	Schrödinger, LLC	https://pymol.org
AlphaFold2	DeepMind	https://github.com/deepmind/alphafold
CellPose 2.0	Stringer et al.	https://www.cellpose.org
CellTool	University of Warsaw	https://celltool.org
ImageJ/FIJI	NIH	https://imagej.net
Imaris	Oxford Instruments	https://imaris.oxinst.com
ZS XPLORER	Malvern Panalytical	Zetasizer Nano ZS
GraphPad Prism	GraphPad Software	https://www.graphpad.com
Adobe Illustrator	Adobe Inc.	https://adobe.com/illustrator
Custom Python scripts for VLP reconstruction and modeling	Maayouf et al^1^	GitHub/Zenodo: https://doi.org/10.5281/zenodo.15579602 (see ref.^12^)

**Other**		

Sylgard™ 184 Silicone Elastomer Kit	Dow	4019862
ChemiDoc Imaging System	Bio-Rad™	12003154
Eppendorf centrifuge 5424 R	Eppendorf	Model: 5424 R
Avanti J-E Centrifuge	Beckman Coulter	Model: Avanti J-E
JA-20 Rotor	Beckman Coulter	Model: JA-20
JLA-10.5 Rotor	Beckman Coulter	Model: JLA-10.5
Fisher Scientific 505 Ultrasonic Sonicator	FisherScientific	12893543
Zetasizer Nano ZS	Malvern Panalytical	ZEN3600
Spinning disk confocal microscope (Ti2e SD CSU W1)	Nikon	/
Confocal microscope (LSM 800)	Zeiss	/
NGC Quest™ 10 Plus Chromatography System	Bio-Rad™	#7880001
CFX Connect™ Real-Time PCR Detection System	Bio-Rad™	1855201
T3000 Thermal Cycler	Biometra(Analytik Jena)	846-X070-070
Mini-PROTEAN® Tetra Cell Electrophoresis System	Bio-Rad™	1658004
Sub-Cell GT Electrophoresis	Bio-Rad™	1704487
4–15% Mini-PROTEAN® TGX Stain-Free™ Protein Gels	Bio-Rad™	4568084
4–15% Mini-PROTEAN® TGX™ Precast Protein Gels	Bio-Rad™	4561083

## Materials and equipment

**Table:** LB Culture Medium

Reagent	Final concentration	Amount
LB Broth Base (Lennox)	1×	20g
NaCl	1×	5g
ddH_2_O	N/A	1L
Total	N/A	1L

***Note:*** Sterilize by autoclaving at 121 °C for 15–20 min. Store at 22°C for up to 1 month.

**Table:** LB Agar

Reagent	Final concentration	Amount
LB Broth Base (Lennox)	1×	20g
NaCl	1×	5g
Agar	1,5%(w/v)	15g
ddH_2_O	N/A	1L
Total	N/A	1L

***Note:*** Sterilize by autoclaving at 121°C for 15–20 min. If not used immediately, molten agar may be maintained at 55–60°C for up to 24–48 h to prevent solidification. For longer storage, pour plates and allow them to solidify. Store plates inverted at 4°C. Antibiotic-free plates may be stored for up to 4 weeks, whereas plates containing ampicillin should preferably be used within 1–2 weeks due to reduced antibiotic stability.

**Table:** Lysis Buffer

Reagent	Final concentration	Amount
NaH_2_PO_4_·H_2_O (MW 137.99 g/mol)	50mM	6.9g
NaCl (MW 58.44 g/mol)	300mM	17.54g
Imidazole (MW 68.08 g/mol)	10mM	0.68g
ddH_2_O	N/A	Add to 1L
Total	N/A	1L

**Preparation note:** Dissolve components in 800 mL ddH_2_O, adjust to pH 8.0 with NaOH, then bring to a final volume of 1 L. Filter-sterilize (0.22 μm) or autoclave if long-term storage is required. Store at 4 °C for up to 3 months.

**Table:** Wash Buffer

Reagent	Final concentration	Amount
NaH_2_PO_4_·H_2_O (MW 137.99 g/mol)	50mM	6.9g
NaCl (MW 58.44 g/mol)	300mM	17.54g
Imidazole (MW 68.08 g/mol)	20mM	1.36g
ddH_2_O	N/A	Add to 1L
Total	N/A	1L

**Preparation note:** Dissolve components in 800 mL ddH_2_O, adjust to pH 8.0 with NaOH, then bring to a final volume of 1 L. Filter-sterilize (0.22 μm) or autoclave if long-term storage is required. Store at 4 °C for up to 3 months.

**Table:** Elution Buffer

Reagent	Final concentration	Amount
NaH_2_PO_4_·H_2_O (MW 137.99 g/mol)	50mM	6.9g
NaCl (MW 58.44 g/mol)	300mM	17.54g
Imidazole (MW 68.08 g/mol)	250mM	17g
ddH2O	N/A	Add to 1L
Total	N/A	1L

**Preparation note:** Dissolve components in 800 mL ddH_2_O, adjust to pH 8.0 with NaOH, then bring to a final volume of 1 L. Filter-sterilize (0.22 μm) or autoclave if long-term storage is required. Store at 4 °C for up to 3 months.

**Table:** SDS-PAGE Running Buffer (1×)

Reagent	Final concentration	Amount
Tris-base	25 mM	3.03g
Glycine	190mM	14.26g
SDS	0,1%(w/v)	1g
ddH_2_O	N/A	Complete to 1L
Total	N/A	1L

**Preparation note:** Store at 22°C for up to 6 months.

## Step-by-step method details

### Design and cloning of peptide-VLP constructs via direct genetic fusion

**Timing: 1–2 weeks (excluding ordering delays)**

This step describes the design and cloning of short bioactive peptide sequences (e.g., RGD, YIGSR) into the AP205 capsid protein gene via direct genetic fusion. Using this approach, the protocol enables generation of both homomeric VLPs displaying a single peptide and heteromeric VLPs formed by co-expression of two differently tagged coat proteins for dual-peptide display.

1. Cloning of Homomeric Peptide-AP205 Constructs: 3–4 days
  ***Note:*** The homomeric AP205 peptide-fusion constructs described in this protocol were cloned into pET15b, which provides an N-terminal His-tag for purification under a T7 promoter. Alternative T7-based expression vectors may also be used, provided they support high-level expression in *E. coli* and include an appropriate affinity tag for purification.
  a. Prepare the coding sequence for the VLP coat protein and bioactive peptide(s).
    ***Note:*** Refer to Tables 1 and 2 for the AP205 coat protein (CP3) sequence and the RGD/YIGSR peptide sequences used in this protocol. If an alternative VLP platform or different peptide sequences are selected, ensure that the coding sequences are codon-optimized for *E. coli* expression.
    **Table 1:** Amino acid and coding DNA sequences of the AP205 coat protein used in this study

Construct	Amino acid sequence	Nucleic acid sequence (5’→3′)
AP205	MANKPMQPITSTANKIVWSDPTRL STTFSASLLRQRVKVGIAELNNVS GQYVSVYKRPAPKPEGCADACVI MPNENQSIRTVISGSAENLATLKA EWETHKRNVDTLFASGNAGLGFL DPTAAIVSSDTTA	ATGGCGAACAAACCGATGCAGCC GATTACCAGCACCGCGAACAAAA TTGTGTGGAGCGATCCGACCCGC CTGAGCACCACCTTTAGCGCGAG CCTGCTGCGCCAGCGCGTGAAAG TGGGCATTGCGGAACTGAACAACG TGAGCGGCCAGTATGTGAGCGTGT ATAAACGCCCGGCGCCGAAACCGG AAGGCTGCGCGGATGCGTGCGTGA TTATGCCGAACGAAAACCAGAGCAT TCGCACCGTGATTAGCGGCAGCGCG GAAAACCTGGCGACCCTGAAAGCGG AATGGGAAACCCATAAACGCAACGT GGATACCCTGTTTGCGAGCGGCAAC GCGGGCCTGGGCTTTCTGGATCCGA CCGCGGCGATTGTGAGCAGCGATA CCACCGCG

**Table 2:** Amino acid sequence of the different peptides and their origin

Peptide name	Sequence	Description
RGD	GRGDSPK	Derived from RGD fibronectin site
YIGSR	GYIGSRGYG	Derived from β1 laminin chain
BMP2	KIPKASSVPTELSAISTLYL	Derived from rh-BMP2 (73 to 92 amino acids)
6x His-tag	HHHHHH	Purification tag
HA-tag	YPYDVPDYA	Derived from human influenza hemagglutinin
  b. Design the fusion construct *in silico* using sequence editing software (e.g., Benchling or SnapGene).
    i. Download the pET-15b vector sequence and define the insertion site (HindIII and NdeI).
    ii. Remove the region between HindIII and NdeI.
    iii. Insert the AP205 coat protein sequence with a C-terminal peptide fusion (RGD or YIGSR), ensuring the N-terminal His-tag is preserved for purification.
    iv. Include a short flexible linker (e.g., GSGS) between the coat protein and the peptide to improve accessibility and reduce potential structural interference.
    v. Ensure correct reading frame and junction sequences.
    vi. Save the final annotated construct and export for primer design.
      **Optional but recommended:** Before finalizing construct design, use AlphaFold2 to model the 3D structure of the peptide-fused capsid protein. This aids in identifying potential structural clashes and ensures that the fused peptide remains surface-exposed. The predicted monomer can be aligned to the VLP scaffold using known capsid structures (e.g., PDB: 5LQP for AP205, PDB: 7B3Y for Mi3). Alignment and reconstruction can be performed using a custom PyMOL script previously developed for this protocol (see GitHub repository,^12^ and Figures 2 and 3 for detailed instructions). This facilitates rational linker placement and supports the structural feasibility of the designed fusion.

**Figure 2:**
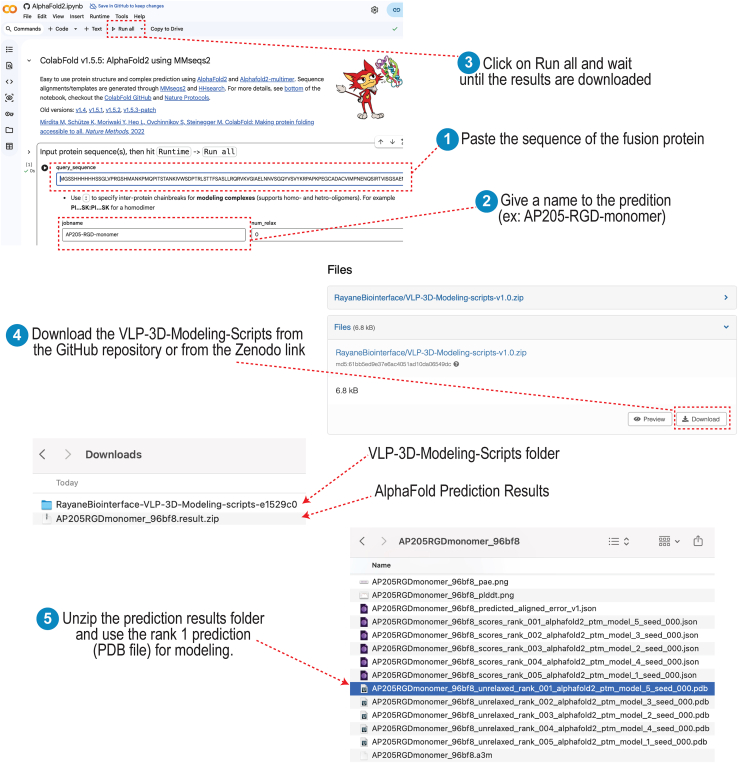
VLP modeling Steps Part 1: Structural prediction of fusion proteins using AlphaFold & modeling scripts downloading The amino acid sequence of the fusion protein (e.g., AP205–peptide fusion) is pasted into the AlphaFold interface and a name is assigned to the prediction. The modeling job is executed and the resulting files are downloaded. The output folder contains several ranked structural models; the highest-confidence model (rank_1 PDB file) is selected as the monomeric structure for downstream VLP reconstruction.

**Figure 3:**
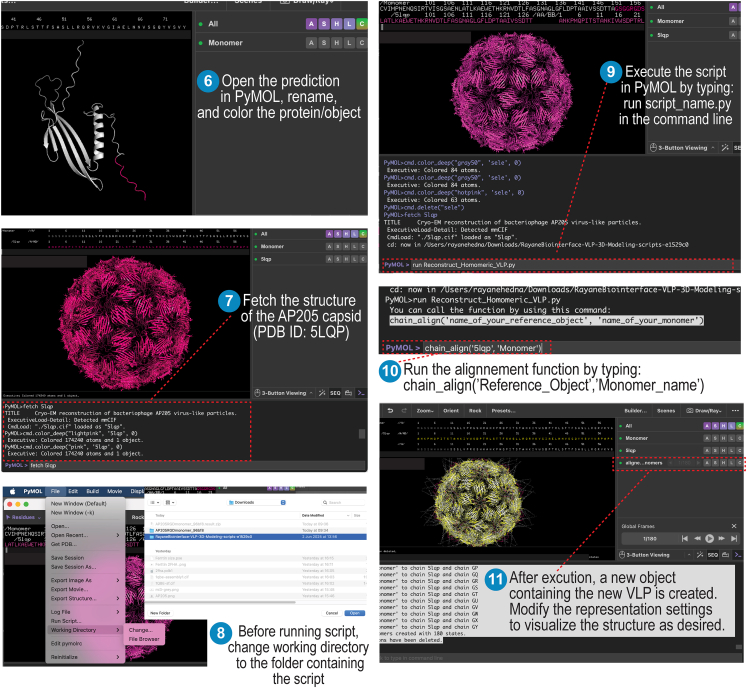
VLP modeling Steps Part 2: Reconstruction of VLP assemblies in PyMOL The predicted monomer structure is opened in PyMOL and prepared for modeling. The reference capsid structure of the AP205 VLP (PDB ID: 5LQP) is retrieved and the working directory is set to the folder containing the reconstruction scripts. The script is executed to align the predicted monomer to the capsid subunits, generating the full VLP assembly. After execution, a new object corresponding to the reconstructed VLP particle is created and can be visualized or rendered using standard PyMOL representation settings.
  c. Design primers for seamless cloning.
    i. For In-Fusion HD (Takara), select 15 bp overlaps with the pET15b linearized vector and 18 -25 bp overlaps with the AP205-peptide insert.
      ***Note:*** See Link for In-Fusion primer design instructions.
    ii. Order primers from a commercial supplier.
      ***Note:*** Primer sequences for constructs used in this protocol are listed in Tables 3 and 4.
      **Table 3:** Primers and plasmids used to clone the different bioactive VLPs by direct genetic fusion

Inserts	Plasmid	Primers (5’→3′)
AP205-RGD	pET15b	Forward: GCCGCGCGGCAGCCATATGGC GAACAAACCGATGCAGC, Reverse: GTTAGCAGCCGGATCCTTATTTC GGGCTATCGCCGCGGCCGCCGC TGCCCGCGGTGGTATCGCTGCTCAC
AP205-BMP2	pET15b	*Forward:* GCCGCGCGGCAGCCATAT GGCGAACAAACCGATGC AGC, Reverse: GTTAGCAGCCGGATCCT TACAGATACAGGGTGCTAATCGC GGACAATTCCGTCGGCACACTCGA GGCTTTTGGGATTTTGCCGCTGCC CGCGGTGGTATCGCTGCTCAC
AP205-YIGSR	pET15b	*Forward:* GCCGCGCGGCAGCCATATGGCGAAC AAACCGATGCAGC, Reverse: GTTAGCAGCCG GATCCTTACCCGTAACCGCGGCTGC CAAT ATAGCCGCTGCCCGCGGTGGTATCGCTGC
HA-AP205-BMP2	pETDuet	*Forward:* AGAAGGAGATATACAATGTATCCGTAT GATGTGCCGGAT TATGCGGGCAGCGGCGGC AGCGGCATGGCGAACAAACC GATGC, Reverse: CCAATTGAGATCTGCTTACAGATACAGGGTGCT AATCGC GGACAATTCCGTCGGCACACTCGAG GCTTTTGGGAT TTTGCCGCTGCCCGCGGTGG TATCGCTGCTCAC
HA-AP205-YIGSR	pETDuet	*Forward:* AGAAGGAGATATACATATGTA CCCATACGATGTTCCAGATTACGC TGGCAGCGGCGCGAACAAACCG ATGCAG, Reverse: GCGTGGC CGGCCGATATCCAATTGAGATCTGCTT ACCCGTAACCGCGGCTGCCAATATAG CCGCCGCTGCC

**Table 4:** Primers and plasmids used to clone the SpyTagged peptides and the mi3 SpyCatcher VLP

Inserts	Plasmid	Primers (5’→3′)
SpyTag-ELP-RGD	pET15b	Forward: GCCGCGCGGCAGCCATATGGCCCATATTG TCATGGTTGATGC, Reverse: GTTAGCAGCCGG ATCCTCTTATTTCGGGCTATCGCCGCGGCCGCCGCTGCCG TCGAGCAGCCCGCCCGGCACG
Mi3 SpyCatcher	pET15b	Forward: CGCGGCAGCCATATGGGTTCAAGTGTAACCACC, Reverse: GCTTTGTTAGCAGCCGGATCCTTATT CTGTACAGCCACGAATTTTTTCC
  d. Once the primers received, amplify the AP205-peptide insert by PCR using high-fidelity DNA polymerase.
    i. Set up a 50 μL PCR reaction according to manufacturer’s instructions.
    ii. Run the PCR product on a 1,2% agarose gel and verify expected size.
      ***Note:*** Adjust gel concentration if needed.
    iii. Purify PCR product using a gel extraction kit (e.g., Qiagen; see Link).
      ***Note:*** Any other PCR clean up kit could be used.
    iv. Quantify DNA concentrations using a Nanodrop (See troubleshooting 1).
  e. Linearize the pET15b vector using HindIII and NdeI restriction enzymes.
    i. Digest 2–5 μg of plasmid with appropriate enzymes according to manufacturer instructions (Link for Invitrogen fast digest restriction enzymes).
    ii. Run the digest on an agarose gel and extract the linearized vector.
    iii. Quantify DNA concentrations using a Nanodrop.
      ***Note:*** PCR products from step 4 and digestion products from step 5 can be performed on the same agarose gel.
  f. Assemble the construct using In-Fusion HD Cloning according to manufacturer’s protocol.
    i. Digest Mix insert and linearized vector at a 2:1 molar ratio with the In-Fusion enzyme mix.
    ii. Incubate for 15 min at 50°C.
  g. Assemble Transform 1μL of the assembled product into chemically competent TOP 10 *E. coli* (Manufacturer’s protocol).
    i. Plate transformed cells on LB agar containing 100 ug/mL ampicillin.
    ii. Incubate overnight at 37°C (See troubleshooting 2).
  h. Screen colonies for correct insertion.
    i. Pick 4-6 colonies and perform colony PCR using flanking primers or restriction digestion of miniprepped plasmids.
      ***Note:*** Refer to Link for the detailed colony PCR protocol. Use a sterile pipette tip to gently touch a single colony and transfer the cells directly into the PCR premix. Mix briefly and proceed with thermal cycling. Load the PCR products directly onto an agarose gel to verify the presence and size of the insert.
    ii. Confirm successful insert by Sanger sequencing.
  i. Store confirmed plasmids at −20°C for downstream expression.

2. Cloning of Heteromeric Peptide-AP205 Constructs: 3–4 days
  ***Note:*** For heteromeric VLP assembly, constructs are cloned into pETDuet-1, which enables co-expression of two coat protein variants from independent multiple cloning sites.
  a. Design two AP205 constructs, in silico, for co-expression in the same plasmid:
    i. Insert an AP205 coat protein fused to peptide 1 (RGD) into MCS1 at the BamHI site.
      ***Note:*** The vector already includes an N-terminal His-tag upstream of this site for purification.
    ii. Insert a second AP205 coat protein fused to peptide 2 (e.g., YIGSR) into MCS2 between the NdeI and EcoRI restriction sites.
      **CRITICAL:** Include an N-terminal HA-tag instead of a His-tag to allow detection of this subunit and confirm heteromeric particles generation.
      ***Note:*** This dual-expression design enables validation of heteromeric VLP assembly. The His-tag permits purification of particles by nickel affinity chromatography, while the second epitope tag (e.g., HA) allows detection of the co-assembled subunit by SDS-PAGE and/or Western blot analysis. Detection of the HA-tag in the His-tag-purified fraction confirms incorporation of both coat protein variants within the same VLP, indicating successful heteromeric assembly.
  b. Design primers for each insert as described for homomeric constructs.
  c. Amplify the two inserts by PCR using previously validated pET15b-based constructs.
  d. Gel purify PCR products and quantify by Nanodrop.
  e. Clone the first insert (AP205-RGD) into MCS1.
    i. Linearize pETDuet-1 using BamHI at MCS1.
    ii. Clone the insert using In-Fusion HD and validate by sequencing.
  f. Clone the second insert (AP205-YIGSR) into MCS2.
    i. Linearize the pETDuet-1-RGD plasmid using NdeI and EcoRI.
      **CRITICAL:** Ensure insert 1 does not contain these sites. Internal sites would lead to unintended digestion and disrupt the first insert during MCS2 cloning.
    ii. Clone and validate the second insert using In-Fusion kit.
    iii. Sequence both MCS1 and MCS2 regions.
  g. Store validated dual-insert plasmids at −20°C for expression of heteromeric VLPs.

### Design and cloning of VLP display constructs via SpyTag-SpyCatcher conjugation

**Timing: 3–4 days (excluding ordering delays)**

This step describes the preparation of ligand constructs for post-assembly functionalization of SpyCatcher-displaying VLPs. SpyTag–fusion ligands can be cloned or sourced depending on availability.

3. Cloning of SpyTag-Functional Protein Constructs: 3–4 days
  ***Note:*** The SpyTag-Functional Protein constructs described in this protocol were cloned into pET15b, which provides an N-terminal His-tag for purification under a T7 promoter. Alternative T7-based expression vectors may also be used, provided they support high-level expression in *E. coli* and include an appropriate affinity tag for purification.
  a. Begin from the template plasmids:
    ***Note:*** For SpyTag-ELP-RGD: SpyTag-ELP-SpyTag plasmid from addgene could be used as a starting template.
  b. Design the fusion construct *in silico:*
    i. Replace the C-terminal SpyTag with the coding sequence of the desired ligand (RGD), joined via a flexible GSGS linker.
    ii. Define insertion site and confirm correct reading frame and linker junction.
  c. Design primers for seamless cloning as described in Major Step 1.
    ***Note:*** Primer sequences for constructs used in this protocol are listed in Table 3-4.
  d. Amplify the modified SpyTag-ELP-RGD insert by PCR using high-fidelity polymerase (See troubleshooting 1)
  e. Linearize the pET15b vector at selected restriction sites.
  f. Assemble the construct using In-Fusion HD Cloning (See troubleshooting 2).
  g. Transform into competent cells and screen clones by colony PCR and sequencing.
    **Note:** SpyTag–fluorescent proteins (e.g., SpyTag–GFP, SpyTag–mKate) were ordered as plasmids but can also be cloned using the same strategy with appropriate templates (e.g., fluorescent protein-expressing plasmids).

4. Cloning of Mi3–SpyCatcher with His-tag: 3–4 days
  a. **Recommended:** Obtain a His-tagged Mi3-SpyCatcher plasmid directly from Addgene for simplified cloning and expression.
  b. If using the SpyCatcher003 variant (sequence-optimized for improved conjugation efficiency), obtain the corresponding plasmid from Addgene and clone it into pET15b or pET28a with an N- or C-terminal His-tag for purification.
  c. Use the standard cloning procedure described in major step 1: *in silico* design, primer design, PCR amplification, vector linearization, In-Fusion assembly, transformation, and sequence verification.

***Note:*** SpyCatcher003 is a sequence-optimized variant of the original SpyCatcher and offers improved conjugation kinetics. The Mi3–SpyCatcher003 construct available from Addgene does not include a His-tag; if purification via immobilized metal affinity chromatography is needed, a His-tag must be genetically inserted. Alternatively, SpyCatcher (original or 003) can also be fused to other VLP platforms such as AP205 via direct genetic fusion for modular ligand display.

### Expression and purification of VLPs and SpyTag fusion proteins

**Timing: 4–5 days**

This step describes the bacterial expression and purification of peptide-functionalized virus-like particles (VLPs) and SpyTag fusion proteins in *E. coli* BL21(DE3) or equivalent strains. His-tagged constructs were purified by nickel affinity chromatography (Ni-NTA), with an additional size exclusion chromatography (SEC) step for VLPs (e.g., AP205, Mi3) to ensure proper assembly and removal of aggregates, whereas SpyTag fusion proteins were typically used directly after affinity purification.

**Figure 4:**
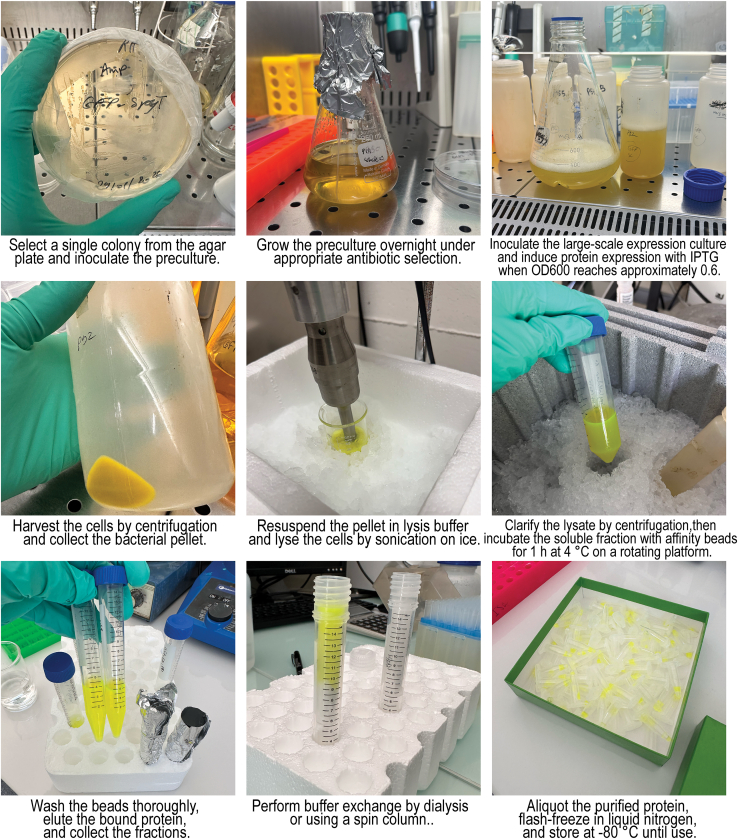
Recombinant protein production and purification workflow Overview of the bacterial expression and purification steps used for producing recombinant GFP.

5. Expression of Constructs (Figure 4): 2days
  a. Transform the expression plasmid into *E. coli* BL21(DE3) using standard heat-shock transformation according to the manufacturer’s instructions (see Link).
  b. Plate on selective LB agar (100 μg/mL ampicillin or 50 μg/mL kanamycin depending on vector) and incubate overnight at 37°C.
  c. Inoculate a 25–50 mL starter culture in LB medium with appropriate antibiotic at 37°C for 18 to 20 h.
  d. Use 1:50–100 dilution in LB medium supplemented with appropriate antibiotic to inoculate large-scale expression culture.
    ***Note:*** Before scaling up to large-volume cultures (e.g., 300–500 mL), it is strongly recommended to perform a small-scale expression test (e.g., 10–50 mL) to verify protein expression and solubility. Analyze the lysate by SDS-PAGE (and Western blot if needed) to confirm successful production of the His-tagged protein. This helps avoid wasting materials on non-expressing or misfolded constructs.
  e. Grow the culture at 37°C until the optical density at 600nm (OD600) reaches 0.6–0.8.
  f. Induce protein expression by adding freshly prepared IPTG (1 mM) and incubate for 4 h at 37°C.
    ***Note:*** Induction temperature and duration can be adjusted depending on construct.
  g. Harvest cells by centrifugation at 4000 × *g* for 15–20 min at 4°C.
  h. Discard supernatant and store the pellets at −20°C (recommended) or proceed directly to lysis.

6. Cell Lysis and Clarification: 2 h
  a. Resuspend pellet in ice-cold lysis buffer (Check buffer list) at 2–5 mL per gram of wet pellet.
  b. Add Lysozyme to a final concentration of 0.5–1 mg/mL and incubate on ice for 30min.
  c. Lyse cells by sonication (6 × 10 s pulses at 200–300 W, with 10 s cooling intervals on ice) or using a French press.
  d. Add DNase I (5 μg/mL), RNase (10 μg/mL) and incubate on ice for 15min.
  e. Clarify lysate by centrifugation at 10,000 × *g* for 20 min at 4°C.
  f. Transfer the clarified supernatant to a fresh tube for purification.

7. Ni-NTA affinity purification (His-tagged proteins): 2–3 h
  a. Incubate the clarified lysate with equilibrated Ni-NTA resin using batch binding for 60 min at 4°C with gentle rotation.
    ***Note:*** As a general guideline, use 1 mL of settled resin per 5–10 mg of expected target protein.
  b. Centrifuge at 500 × *g* for 5 min and discard unbound supernatant.
  c. Wash resin three times with 10 resin volumes of wash buffer (see Buffer List).
  d. Elute bound protein with 4 resin volumes of elution buffer (see Buffer List).
    ***Note:*** A second elution step may be performed using a smaller volume of elution buffer to maximize protein recovery while minimizing sample dilution. Elution fractions can subsequently be pooled.
  e. Collect elution fractions, keep on ice, and analyze by SDS-PAGE.
  f. Combine elution fractions containing the target protein.
  g. Perform dialysis or buffer exchange (e.g., desalting column or dialysis membrane) to remove imidazole prior to downstream applications.
    ***Note:*** For VLP samples, this dialysis step can be skipped, as imidazole will be removed during subsequent size exclusion chromatography.

8. Size exclusion chromatography (SEC)- For VLPs: 2–3 h
  a. Equilibrate the SEC column:
    i. Use an appropriate analytical SEC column such as SuperdexTM 200 Increase 10/300 GL (Cytiva) or Sephacryl® S-200 HR (Sigma-Aldrich).
    ii. Equilibrate the column with sterile-filtered 1x PBS (pH 7.4) at the recommended flow rate (typically 0.5–0.75 mL/min).
      ***Note:*** All SEC runs in this protocol were performed using the NGS Bio-Rad chromatography system equipped with UV detection and automated fraction collection.
  b. Prepare and inject the VLP sample directly onto the column.
    ***Note:*** Typical injection conditions used in this study were 100 μL of VLP sample at an approximate concentration of 600 μg/mL. Injection volume and concentration may be adjusted depending on SEC column specifications.
    ***Optional:*** Concentrate VLPs using Amicon Ultra-15 filters, 100 kDa cutoff, if needed.
  c. Run and monitor elution at a constant flow rate according to column specifications.
  d. Collect fractions corresponding to the VLP peak (typically 13-15 mL for AP205 or Mi3) and analyze by SDS-PAGE (See troubleshooting 3, troubleshooting 4 & troubleshooting 5).
  e. Quantify proteins:
    i. Recommended: Use Micro BCA Protein Assay (see Link).
    ii. Alternatively, measure absorbance at 280 nm using a Nanodrop with appropriate extinction coefficient.
      ***Note:*** SEC can be used in either an analytical or preparative mode. Analytical SEC (e.g., Superdex 200 Increase 10/300, Sephacryl® S-200 HR 24 mL) is suitable for confirming VLP assembly, size distribution, and purity. Preparative SEC (e.g., Superdex 200 pg, Sephacryl® S-400 HR) offers higher loading capacity and is better suited for large-scale purification of VLPs for downstream applications (see Link). Choose the column type based on experimental goals and required sample yield.

### SpyTag-SpyCatcher conjugation and VLP characterization by DLS and TEM

**Timing: 1–2 days**

This step details the covalent conjugation of SpyTag-fused ligands (e.g., SpyTag–ELP–RGD, SpyTag–fluorescent proteins) to SpyCatcher-displaying Mi3 VLPs, followed by characterization of VLP integrity and size distribution using dynamic light scattering (DLS) and transmission electron microscopy (TEM). These analyses are also applicable to genetically fused AP205-based VLPs for assessing particle assembly and homogeneity.

9. Conjugate SpyTag Ligands to SpyCatcher–Displaying VLPs: 4 h
  a. Determine the molar concentrations of VLPs and SpyTag-fused ligands using protein concentrations obtained from the BCA assay and the known molecular weights of each species.
    ***Note:*** For VLPs, estimate the effective molar mass by multiplying the molecular weight of a single capsid subunit by the total number of subunits per particle (180 for AP205 VLPs and 60 for Mi3–SpyCatcher VLPs). This approximation allows estimation of the molar concentration of intact VLP particles, which is required to define accurate ligand-to-VLP stoichiometry during conjugation.
  b. Define ligand-to-SpyCatcher molar ratios to be tested (e.g., 0.25:1, 1:1, 2:1, 3:1), depending on the desired degree of functionalization.
  c. Prepare 20**–**50 μL reaction mixtures in PBS using calculated volumes to achieve the selected molar ratios.
  d. Incubate reactions at 25°C for 45 min with continuous shaking at 400 rpm.
  e. Analyze conjugation efficiency by SDS-PAGE.
    i. Load reaction samples alongside appropriate controls, including SpyCatcher-VLPs alone and SpyTag-ligand alone.
    ii. Confirm covalent conjugation by the appearance of higher molecular weight bands corresponding to VLP-ligand complexes.
    iii. Select the optimal ligand-to-VLP ratio as the condition where no free SpyTag-ligand and no unconjugated SpyCatcher subunits are visible, indicating full saturation of available binding sites (See troubleshooting 6).
  f. For dual-ligand conjugation:
    i. Prepare a mixture of two different SpyTag-ligands at desired ratios, and repeat the incubation step.
    ii. Verify dual conjugation by the presence of multiple shifted bands on SDS-PAGE.
      ***Note:*** The approaches described here estimate average ligand occupancy across the VLP population and do not directly verify co-display on individual VLPs. For additional validation of dual conjugation on the same particle, orthogonal strategies such as immunoprecipitation using epitope-tagged SpyTag ligands (e.g., HA-tagged ligands) followed by detection of the second ligand may be implemented.

10. Size estimation by DLS and conjugation validation for SpyTag SpyCatcher VLPs: 2 h
  a. Resuspend Prepare samples of both conjugated and unconjugated VLPs at a final concentration of 0.1 mg/mL in sterile 1x PBS.
  b. Clarify each sample by brief centrifugation and filtration through a 0.22 um low-protein-binding syringe filter to remove aggregates and particulate debris.
  c. Measure the hydrodynamic diameter using a dynamic light scattering (DLS) instrument at 25°C under standard conditions.
  d. Evaluate conjugation efficiency by comparing the hydrodynamic diameter of conjugated VLPs to the unconjugated control. A reproducible increase in particle size indicates successful ligand attachment to the VLP surface.

***Note:*** DLS is not recommended for analyzing VLPs conjugated to fluorescent proteins (e.g., SpyTag–GFP or SpyTag–mKate), as fluorophores interfere with light scattering and can yield inaccurate size measurements.

11. Transmission Electron Microscopy (TEM): 1 hour for preparation
  a. Apply 5**–**10μL of purified VLP sample onto glow-discharged, carbon-coated 400-mesh copper grids.
  b. Incubate for 1 minute, then gently blot excess liquid using filter paper.
  c. Stain the grid with 2% (w/v) uranyl acetate for 30 seconds at 22°C.
  d. Blot again and air-dry the grid completely before imaging.
  e. Visualize the VLPs using a transmission electron microscope.
  f. Assess particle morphology and structural integrity, comparing conjugated and unconjugated samples to verify preservation of capsid architecture after functionalization.

***Note:*** The characterization procedures described above, dynamic light scattering (DLS) and transmission electron microscopy (TEM), are equally applicable to VLPs produced by direct genetic fusion of peptides. These analyses enable assessment of particle size, homogeneity, and structural integrity regardless of the functionalization strategy used.

### Surface functionalization of PDMS using virus-like particles

**Timing: 1–2 days**

**Figure 5:**
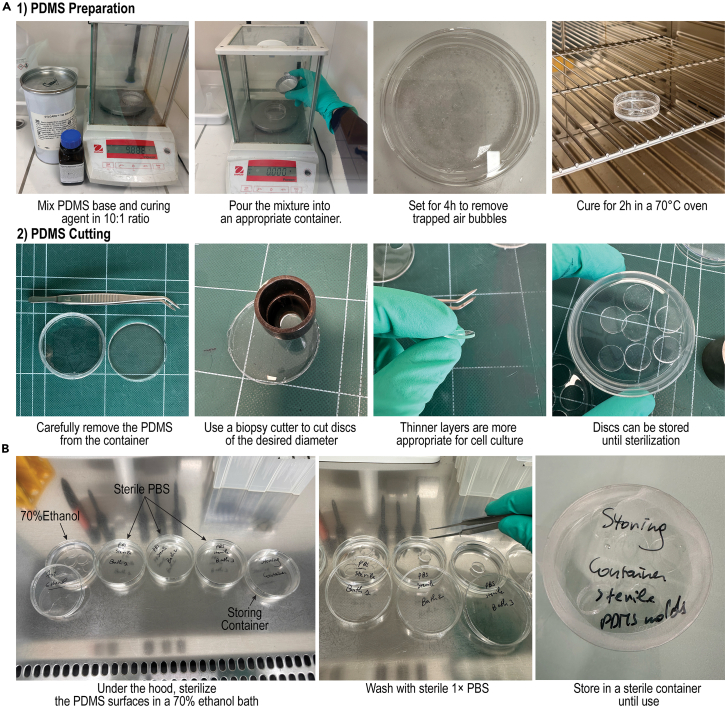
Preparation and sterilization of PDMS substrates (A) PDMS surfaces preparation. (B) Sterilization of PDMS discs.

This step describes the preparation of PDMS substrates (Figure 5) and their functionalization via passive adsorption of virus-like particles (VLPs).

12. Preparation of PDMS Substrates: 1 day
  a. Mix the PDMS base and curing agent at a 10:1 (w/w) ratio in a disposable container
  b. Transfer around 2**–**4 g of the mixture into 60 mm polystyrene Petri dishes and spread evenly across the surface to ensure uniform thickness.
  c. Allow the PDMS to degas at 25°C for 4 h to eliminate entrapped air bubbles.
    ***Note:*** Passive degassing at ambient pressure is sufficient. Alternatively, vacuum degassing may be used (10**–**15 min under reduced pressure) to accelerate bubble removal.
  d. Cure the degassed PDMS by transferring the dishes to a dry oven at 70°C for 2 h.
  e. Cut the cured PDMS into discs of the desired diameter (e.g., 10-15 mm) using a biopsy punch.
  f. Sterilize the PDMS substrates by immersion in 70% ethanol for 15 min, followed by three rinses with sterile deionized water. Allow to dry completely under sterile conditions.
  g. Store the sterilized PDMS substrates in closed sterile containers at 25°C until use (Figure 5).
    ***Note:*** PDMS was selected because it is inherently bioinert, cells do not adhere to it in the absence of surface modification.^13^ This makes it an ideal blank substrate for evaluating the biological effects of VLP-based functionalization.

13. Surface saturation with pre-functionalized VLPs: 2 h
  a. Prepare VLP suspensions in sterile 1× PBS at a range of concentrations (e.g., 10, 25, 50, 100, and 200 μg/mL).
  b. Add 50 to 100μL of each VLP solution to fully cover the surface of each PDMS disc.
  c. Incubate the plates at 25°C for 30**–**40 min without agitation to allow adsorption.
  d. Remove the VLP solution and gently wash each disc three times with x mL of sterile 1× PBS to eliminate unbound particles.
  e. Label surface-bound VLPs using anti-His tag immunostaining:
    i. Cover each PDMS surface with anti-6×His tag mouse monoclonal antibody diluted 1:1000 in PBS containing 1% BSA, and incubate for 1 hour at 25°C.
      ***Note:*** PBS containing 1% BSA is used as a blocking buffer to minimize nonspecific antibody adsorption to the PDMS substrate during immunostaining. This step improves confidence that the detected anti-His fluorescence signal originates from surface-bound VLPs rather than nonspecific interactions between antibodies and the substrate.
    ii. Wash surfaces 3 times with PBS to remove unbound primary antibody.
    iii. Incubate with secondary antibody diluted 1:500 in PBS with 1% BSA for 45 min at 25°C, protected from light.
    iv. Wash surfaces 3 times with PBS to remove unbound secondary antibody.
  f. Acquire fluorescence images using a confocal laser scanning microscope (e.g., Zeiss LSM 800) using excitation/emission settings appropriate for the selected fluorophore (See troubleshooting 7).
    ***Note:*** In our experiments, Alexa Fluor™ 647-conjugated secondary antibodies were imaged using excitation/emission settings appropriate for the far-red channel. Imaging was performed using a 63× water objective to obtain sufficient spatial resolution for visualization of VLP surface coating, although other objectives and acquisition settings may be used depending on the microscope configuration, desired field of view, and resolution.
  g. Quantify fluorescence intensity using FIJI/ImageJ from five representative images per sample, across three independent experiments (n = 3).
    i. Calculate the mean fluorescence intensity per surface.
    ii. Subtract background fluorescence measured from BSA-treated PDMS control surfaces processed using the same immunostaining procedure.
  h. Plot fluorescence intensity versus VLP concentration and fit the data to a nonlinear binding model to determine the surface saturation point.
  i. Use the determined saturation concentration to prepare VLP-functionalized PDMS surfaces for subsequent cellular assays.

14. On-Surface Ligand Conjugation for SpyCatcher–Displaying VLPs: 1 hour
  ***Note:*** This step applies when using VLPs that display surface-accessible SpyCatcher domains (e.g., Mi3–SpyCatcher). Following adsorption of the VLPs onto the substrate, SpyTag-functionalized ligands can be covalently attached directly on the surface, enabling modular post-functionalization of pre-adsorbed VLP coatings without requiring preparation and adsorption of newly functionalized VLPs. This strategy may be advantageous for ligands that alter VLP surface properties, such as charge, and thereby affect adsorption efficiency when pre-displayed on the VLP surface. In addition, performing ligand conjugation after VLP immobilization may help avoid aggregation issues that can arise with certain peptide-displaying VLP formulations while maintaining controlled ligand orientation through SpyTag–SpyCatcher coupling.
  a. Adsorb SpyCatcher-VLPs onto PDMS substrates at the previously determined saturation concentration (as described in Step 4.2).
  b. Wash surfaces three times with sterile PBS to remove unbound VLPs.
  c. Incubate the surfaces with a SpyTag-fused ligands solution (e.g., SpyTag-RGD, SpyTag-GFP, SpyTag-mKate) for 10 min at 25°C in PBS. Use sufficient volume to fully cover the surface.
  d. Wash surfaces three times with sterile PBS to remove unbound ligands.
    ***Note:*** Because the substrate is already coated with adsorbed VLPs prior to ligand incubation, exposure of the underlying PDMS surface is reduced, thereby decreasing the likelihood of nonspecific adsorption of SpyTag-fused ligands. In addition, ligand incubation was performed using low micromolar concentrations (e.g., 1.5 μM SpyTag-GFP) and short incubation times (10 min), conditions sufficient to promote efficient SpyTag–SpyCatcher conjugation while further limiting nonspecific adsorption to the surface.

15. On-Surface Ligand Conjugation of multiple SpyTag ligands: 1 hour
  a. Adsorb SpyCatcher-VLPs onto PDMS substrates at the previously determined saturation concentration (as described in Step 4.2).
  b. Wash surfaces three times with sterile PBS to remove unbound ligands.
  c. Prepare an equimolar mixture of two SpyTag-fused ligands (e.g., SpyTag-GFP and SpyTag-mKate) in PBS.
  d. Incubate the surfaces with the ligands for 10**–**15 min at 25°C in PBS.
  e. Wash surfaces three times with sterile PBS to remove unbound ligands.
  f. For controlled stoichiometry, adjust the input ratio of the SpyTag ligands according to the desired surface composition.
    ***Note:*** For more precise control over ligand ratios, perform conjugation in solution (see Step 4: SpyTag–SpyCatcher Conjugation), then adsorb the pre-conjugated VLPs onto PDMS
  g. Validate co-display using fluorescence microscopy (for fluorescent ligands) or immunostaining with orthogonal epitope tags (for non-fluorescent proteins or peptides).
    ***Note:*** In this on-surface conjugation strategy, the input ratio of SpyTag ligands primarily influences the average ligand composition across the VLP-coated surface rather than the exact composition of individual VLPs.

### Functional validation assays on VLP-functionalized surfaces

**Timing: 1–2 days**

This This step describes the seeding of C2C12 cells onto VLP-functionalized PDMS substrates and the assessment of cell adhesion and early spreading responses induced by surface-displayed peptides.

***Note:*** The functional validation assays implemented in this step are selected based on the identity and expected biological activity of the peptide displayed on the VLP surface. In this protocol, cell adhesion and early spreading are used as primary readouts. Additional functional assays may be implemented depending on the selected bioactive peptide and intended biological application. Examples including cell differentiation and migration assays are described in our associated primary research paper.^1^

16. Cell Preparation: 30min
  a. Culture adherent cells C2C12 cells (or other) in complete growth medium at 37°C and 5% CO2.
  b. Maintain cells below 80% confluence.
  c. Detach cells using trypsin-EDTA according to standard protocols.
  d. Neutralize enzymatic activity, collect cells, and centrifuge at 300 x g for 5 min.
  e. Resuspend the pellet in serum-free or low-serum medium to minimize nonspecific protein adsorption.
  f. Count cells and adjust to the desired seeding density.

17. Cell Seeding on VLP-Functionalized Surfaces: 30min
  a. Rinse each substrate once with sterile PBS and once with pre-warmed culture medium.
  b. Seed cells at a defined density (typically 5,000**–**10,000 cells/cm^2^).
    ***Note:*** Include the following controls: Bare PDMS (negative adhesion control), PDMS functionalized with non-bioactive or non-functionalized VLPs and PDMS coated with soluble ECM protein (e.g., fibronectin), if relevant.
  c. Incubate at 37 °C for 4 to 18 h.

18. Assessment of Cell Adhesion: 1 h
  a. After 4-18 h of incubation, gently wash substrates once with warm PBS to remove non-adherent cells.
  b. Fix adherent cells using 4% paraformaldehyde for 15**–**20 min at 25°C.
  c. Wash three times with PBS.
  d. Permeabilize cells with 0.1% Triton X-100 in PBS for 5 min.
  e. Block nonspecific binding of staining reagents using 1% BSA in PBS for 30 min.
  f. Stain the actin cytoskeleton using fluorescent phalloidin and nuclei using DAPI.
  g. Acquire fluorescence images using confocal microscopy under identical acquisition settings across all conditions (See troubleshooting 8).

19. Quantification and Data Analysis: 2 h
  a. Quantify cell adhesion and spreading from fluorescence microscopy images following F-actin and nuclear staining. Individual cells are segmented using the CellPose 2.0 plugin in FIJI/ImageJ with a minimum cell area threshold of 100 μm^2^.
    ***Note:*** Cell spreading is quantified as the projected cell area (μm^2^) for each segmented cell. Where indicated, basic morphology descriptors (e.g., circularity) are extracted using FIJI/ImageJ or CellTool for descriptive analysis.
  b. For each condition, analyze ‚80 cells from randomly selected fields of view, pooled from at least three independent experiments.
  c. Report adhesion and spreading metrics as mean ± standard deviation.

## Expected outcomes

**Figure 6:**
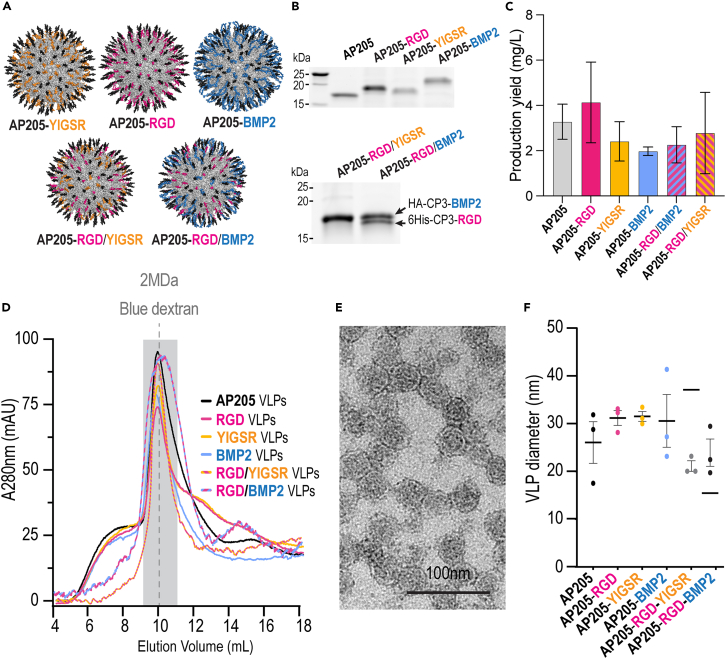
Production and structural characterization of engineered AP205 VLPs (A) Modeled representations of the different AP205 direct genetic fusion VLP constructs generated using this protocol, with rendering performed as described in Figures 2 and 3. (B) SDS–PAGE analysis confirming expression of the peptide-fused AP205 coat protein variants. (C) Production yield obtained for each construct following recombinant expression and purification in *E. coli*. (D) Size-exclusion chromatography (SEC) profiles showing elution of assembled VLPs at high molecular weight, near the 2 MDa blue dextran reference peak. (E) Representative transmission electron microscopy (TEM) image confirming the formation of spherical VLP particles. Scale bar: 100 nm. (F) Dynamic light scattering (DLS) analysis showing the hydrodynamic diameter of assembled VLP particles in solution.

This protocol enables the production of peptide- or protein-displaying virus-like particles (VLPs) and their use for biomaterial surface biofunctionalization through high-copy presentation of peptides or proteins on the VLP surface. Recombinant expression of AP205 peptide-fused constructs or SpyTag-fusion proteins in *E. coli* typically yields assembled VLPs with purification yields in the range of approximately 1–4 mg per liter of bacterial culture following Ni-NTA purification and size exclusion chromatography (Figures 6B and 6C). Successful particle assembly is confirmed by size exclusion chromatography profiles showing a dominant VLP peak and by transmission electron microscopy images revealing uniform nanoscale particles with intact capsid morphology (Figures 6D and 6E). Dynamic light scattering (DLS) can provide an approximate estimate of particle hydrodynamic diameter but may not always resolve a fully homogeneous VLP population, as the technique is sensitive to small aggregates and larger particles that disproportionately influence the scattering signa (Figure 1F).

**Figure 7:**
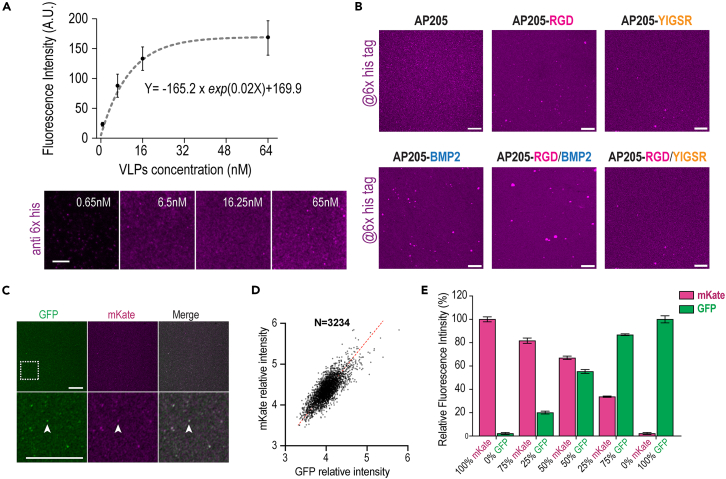
PDMS surface functionalization using VLPs and modular ligand display (A) Quantification of AP205-RGD adsorption on PDMS surfaces using anti-6×His immunofluorescence. Increasing VLP concentration during adsorption leads to a corresponding increase in fluorescence intensity until surface saturation is reached, enabling determination of the optimal concentration for surface coating (65nM). Representative fluorescence images of surfaces incubated with increasing VLP concentrations are shown below the graph. (B) Representative fluorescence microscopy images of PDMS substrates coated with different AP205 genetic fusion VLP constructs under saturation conditions and detected by anti-6×His immunostaining following adsorption. The same coating protocol and fluorescence intensity analysis described in panel A were performed for all VLP conditions. (C) Confocal fluorescence images of PDMS substrates coated with Mi3-SpyC VLPs following conjugation with SpyTag-fused GFP and mKate mixed at a 1:1 ratio. Insets show magnified regions highlighting overlapping fluorescence signals from both reporters on the surface. Scale bars: 10 μm. (D) Correlation analysis of GFP and mKate fluorescence signals detected from individual surface features. Local fluorescence maxima were identified using the Find Maxima function in ImageJ, and the corresponding GFP (x-axis) and mKate (y-axis) intensities were plotted for each detected feature. (E) Relative fluorescence intensities of GFP and mKate measured at different input ratios of SpyTag-fused ligands, demonstrating tunable co-display of multiple ligands on the VLP-functionalized PDMS surface.

For constructs generated by direct genetic fusion (Figure 6A), most short peptides (e.g., RGD or YIGSR) are expected to be well tolerated by the AP205 scaffold and yield homogeneous particles. In some cases, larger or structurally constrained peptide domains may reduce assembly efficiency or induce partial aggregation, which can appear as broader peaks during size exclusion chromatography or heterogeneous particle populations in DLS and TEM analyses. For example, the BMP-2 mimetic peptide tested in our primary study^1^ showed a tendency to aggregate at higher concentrations and did not produce functional particles under the tested conditions (Figure 7B). In such cases, buffer optimization (e.g., pH and salt concentration), redesign of the peptide sequence, or the use of alternative display strategies such as SpyTag–SpyCatcher-mediated conjugation may improve particle stability and ligand presentation.

Following adsorption onto PDMS substrates, VLPs form a uniform protein coating that can be detected by anti-His immunostaining and fluorescence microscopy (Figures 7A and 7B). Increasing the concentration of VLPs during adsorption produces a corresponding increase in fluorescence intensity until surface saturation is reached, allowing determination of the optimal concentration for surface functionalization (Figure 7A). When SpyCatcher-displaying VLPs are used, SpyTag-fused ligands can be conjugated either prior to adsorption or directly on the VLP-coated surface. In both configurations, the platform enables modular and tunable presentation of peptides or proteins by conjugating distinct SpyTag-fused ligands onto the same SpyCatcher-VLP scaffold without requiring re-engineering of the VLP. When two SpyTag-fused fluorescent reporters, such as GFP and mKate, are applied simultaneously, dual-color fluorescence can be observed, and the relative signal intensities reflect the input ligand ratio, demonstrating controlled averaged co-display on the VLP-coated substrate (Figures 7C–7E). In addition, maxima-based fluorescence correlation analysis of the 50:50 GFP/mKate condition revealed spatially correlated signals, supporting co-localized presentation of both ligands on the surface and consistent with co-display on the same VLP particles.

**Figure 8:**
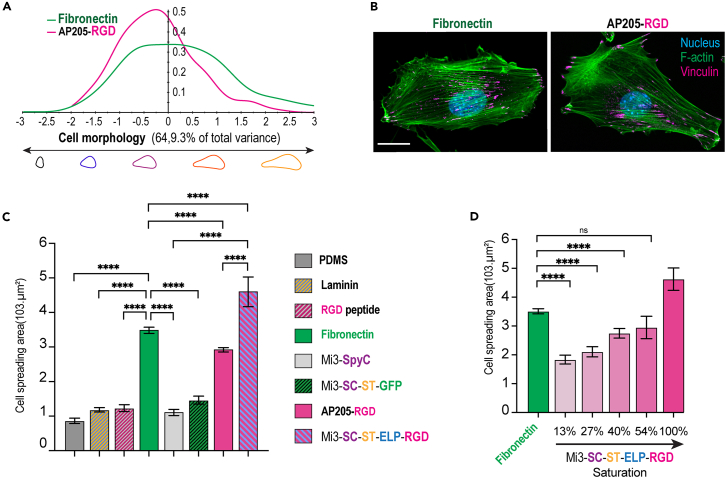
Cell adhesion and spreading on VLP-functionalized PDMS substrates (A) Quantitative analysis of cell morphology showing the distribution of cell shape descriptors for cells cultured on fibronectin-coated surfaces compared with AP205-RGD VLP-coated substrates. The principal morphology axis captures 64.93% of the total variance in cell shape. (B) Representative fluorescence microscopy images of cells cultured on fibronectin or AP205-RGD VLP-coated PDMS substrates. Cells are stained for F-actin (green), vinculin (magenta), and nuclei (blue), revealing formation of actin stress fibers and focal adhesions on both surfaces Scale bar: 30 μm. (C) Quantification of cell spreading area on PDMS substrates functionalized with different coatings, including laminin, soluble RGD peptide, fibronectin, Mi3-SpyC VLPs, Mi3-SpyC–GFP, AP205-RGD VLPs, and Mi3-SpyC–ELP-RGD constructs. Results demonstrate enhanced cell spreading on VLP-based RGD-presenting substrates compared with non-functionalized controls. (D) Cell spreading area measured as a function of ligand density on Mi3-SpyC–ELP-RGD VLP-coated substrates, showing a dose-dependent increase in spreading that approaches the levels observed on fibronectin-coated controls.

Functionalized surfaces are expected to modulate cellular behavior according to the identity of the displayed ligand. For example, surfaces displaying integrin-binding peptides such as RGD typically promote increased cell adhesion and spreading compared with non-functionalized PDMS controls and free RGD peptides (Figure 8). Free RGD peptides alone are generally insufficient to induce efficient cell spreading on PDMS due to limited adsorption stability and suboptimal ligand presentation. The use of VLPs as multivalent scaffolds enables stable surface association and high-density presentation of RGD motifs, thereby improving integrin receptor engagement and downstream cell adhesion responses. Using the SpyCatcher system, the density of RGD on VLPs surface can be tuned, enabling modulation of cell adhesion responses (Figure 8D). Interestingly, differences were also observed between AP205-RGD and MI3-SC-ST-ELP-RGD coatings, suggesting that the ELP component may enhance ligand accessibility and flexibility while additionally contributing mechanical and structural properties to the surface coating. In addition to high-density ligand presentation, VLPs provide a structurally defined nanoscale scaffold that enables multivalent clustering and localized presentation of bioactive motifs, features that may enhance receptor organization and signaling compared with conventional surface tethering approaches. Furthermore, the modular nature of the SpyTag/SpyCatcher system enables the incorporation and co-display of multiple bioactive molecules, including structurally complex proteins that are difficult to present through direct peptide immobilization alone. Finally, because VLPs are recombinant and self-assembling systems, they provide a scalable and reproducible platform for engineering biofunctionalized surfaces with controlled ligand presentation properties. Together, these outcomes confirm that VLP-based surface functionalization provides a robust and reproducible platform for presenting bioactive ligands at high density on biomaterial substrates.

## Limitations

This protocol provides a versatile strategy for biofunctionalizing surfaces using peptide- and protein-displaying virus-like particles (VLPs), but several limitations should be considered. A key constraint is that surface functionalization relies on passive adsorption of VLPs rather than covalent immobilization. While adsorption onto PDMS is reproducible and stable under standard culture conditions, partial desorption or lateral redistribution of VLPs may occur over time, particularly under prolonged incubation or flow. For applications requiring long-term stability or dynamic environments, covalent coupling strategies may be preferable. For applications requiring long-term stability or use in dynamic environments, covalent coupling strategies may be preferable. These may include amide formation, Schiff base chemistry, and other selective covalent immobilization approaches, depending on the biomaterial surface chemistry.^14^^,^^15^

The compatibility between the selected ligand and the VLP platform can also impose constraints. Genetic fusion of large or structured ligands to the capsid protein may impair VLP assembly, reduce expression yields, promote aggregation, or lead to inclusion body formation due to misfolding or poor solubility. Post-assembly functionalization via SpyTag–SpyCatcher chemistry offers a modular solution to these challenges, enabling covalent attachment of ligands after particle assembly. However, this approach requires additional steps, including separate expression of ligands and optimization of conjugation conditions. Precise control of ligand-to-VLP stoichiometry is essential to avoid incomplete conjugation or excess unbound ligand; otherwise, additional purification steps such as size-exclusion chromatography or ultrafiltration are needed, increasing complexity and batch variability.

Furthermore, the adsorption and saturation behavior described here has been optimized for PDMS and validated on titanium, but may not directly translate to other substrate chemistries without re-optimization. Environmental factors such as buffer composition, temperature, and handling may influence VLP stability and surface binding efficiency. Finally, while the protocol allows relative quantification of surface-bound VLPs via immunofluorescence, it does not provide absolute measurements of ligand density or orientation, which may be important for studies focused on receptor clustering or mechanotransduction.

## Troubleshooting

### Problem 1 (steps 1 and 2)

No PCR product or weak amplification.

### Potential solution

This may result from incorrect primer design, suboptimal annealing temperature, or poor template quality. Verify primer sequences and ensure correct overlap regions for the cloning strategy. Perform a gradient PCR to optimize annealing temperature and confirm template integrity. Increasing template concentration or using a high-fidelity polymerase optimized for GC-rich templates may improve amplification efficiency.

### Problem 2 (steps 1 and 2)

Cloning is unsuccessful or few colonies are obtained after transformation.

### Potential solution

This may occur due to inefficient ligation/assembly or incomplete vector digestion. Confirm the integrity and concentration of both insert and vector, and verify that the vector backbone has been fully linearized. Increasing the insert-to-vector molar ratio or repeating the assembly reaction with freshly prepared reagents may improve cloning efficiency.

### Problem 3 (step 3)

After cloning, the plasmid sequence is correct, but the bacteria do not express the protein.

### Potential solution

This issue may be due to toxicity of the expressed protein, poor solubility leading to inclusion body formation, or suboptimal expression conditions. Try reducing the induction temperature (e.g., 18–25°C), lowering IPTG concentration, or switching to a different *E. coli* strain to improve codon usage. Additionally, assess the soluble and insoluble fractions to confirm whether the protein is accumulating in inclusion bodies. In some cases, expression can be improved by using an alternative expression vector such as pET28a, which may provide improved expression depending on tag orientation or vector context.

### Problem 4 (step 3)

After cloning, the bacteria express a truncated version of the protein.

### Potential solution

Truncated protein formation may result from premature translation termination, proteolytic degradation, unstable fusion constructs, or inefficient expression of repetitive or structurally complex sequences. This issue can often be identified by the presence of unexpected lower molecular weight bands during SDS-PAGE analysis. To minimize truncation, verify construct integrity by sequencing prior to expression, reduce induction temperature (e.g., 18–25°C), lower IPTG concentration, and optimize expression duration to reduce proteolytic stress. Using protease-deficient or codon-optimized E. coli strains may also improve expression stability. In addition, western blot analysis using tag-specific antibodies can help confirm whether full-length proteins are being produced.

### Problem 5 (step 3)

Genetically fused VLPs aggregate after expression.

### Potential solution

Fusion of large or structured peptides can interfere with VLP assembly and solubility. In such cases, switching to a post-assembly conjugation strategy using SpyTag–SpyCatcher chemistry is recommended, as it allows modular attachment of ligands without compromising VLP self-assembly.

### Problem 6 (step 4)

Residual free SpyTag–ligand remains after conjugation to SpyCatcher–VLP.

### Potential solution

Excess ligand relative to available SpyCatcher sites can lead to incomplete conjugation. Optimize the ligand-to-VLP ratio to minimize excess, and purify the conjugated VLPs using size exclusion chromatography (SEC) or ultrafiltration to remove unreacted ligands.

### Problem 7 (step 5)

Aggregation occurs during the adsorption of VLPs onto PDMS surfaces.

### Potential solution

This may result from pre-existing aggregates in the VLP preparation or high local concentrations. To avoid this, centrifuge the VLP solution briefly before use and consider filtering through a 0.22 μm low-protein-binding filter to remove aggregates.

### Problem 8 (step 6)

Cells show weak or no response to functionalized surfaces.

### Potential solution

This may indicate that the ligand density is below the biological threshold, or that the ligand is not properly oriented or is inactive in the displayed format. As a control, test the peptide or protein alone to confirm its bioactivity. Additionally, consider testing N-terminal versus C-terminal display formats, and verify ligand accessibility using fluorescence imaging or antibody labeling.

## Resource availability

### Lead contact

Further information and requests for resources should be directed to and will be fulfilled upon reasonable request by the lead contact, Laurent Pieuchot (laurent.pieuchot@uha.fr).

### Technical contact

Technical questions on executing this protocol should be directed to and will be answered by the technical contact, Rayane Hedna (rayane.hedna@uha.fr).

### Materials availability

Plasmid constructs generated in this study are available from the corresponding authors upon request.

### Data and code availability

All data reported in this study are available from the lead contact upon reasonable request.

## Acknowledgments

We gratefully acknowledge the staff operating the 10.13039/100021091IS2M platforms, the 10.13039/501100001665Agence Nationale de la Recherche (grant: SPYMAT ANR-23-CE06-0026), the 10.13039/501100004794Centre National de la Recherche Scientifique, and the 10.13039/501100002717Ministère de l'Enseignement Supérieur et de la Recherche for their financial support. Some elements of the graphical abstract were created using BioRender.

## Author contributions

R.H. performed the experiments, analyzed the data, and wrote the manuscript. H.M. and T.D.S. performed the experiments and analyzed the data. L.P. conceived the study, secured funding, and reviewed the protocol and manuscript.

## Declaration of interests

The authors declare no competing interests.

## Declaration of generative AI and AI-assisted technologies in the writing process

During the preparation of this work, the authors used AI-assisted technologies to assist with language refinement, text editing, and improvement of scientific writing clarity. All generated content was critically reviewed and edited by the authors, who take full responsibility for the final content of the publication.
